# Predicting liver regeneration following major resection

**DOI:** 10.1038/s41598-022-16968-9

**Published:** 2022-08-04

**Authors:** Karolin Dehlke, Linda Krause, Silvana Tyufekchieva, Anastasia Murtha-Lemekhova, Philipp Mayer, Artyom Vlasov, Ursula Klingmüller, Nikola S. Mueller, Katrin Hoffmann

**Affiliations:** 1 Department of General, Visceral and Transplant Surgery, Ruprecht Karls University, Im Neuenheimer Feld 110, 69120 Heidelberg, Germany; 2 Institute of Medical Biometry and Epidemiology, University Medical Center Hamburg-Eppendorf, 20246 Hamburg, Germany; 3 Institute of Computational Biology, Helmholtz Center Munich, Ingolstädter Landstr. 1, 85764 Neuherberg, Germany; 4 Department of Diagnostic and Interventional Radiology, Ruprecht Karls University, 69120 Heidelberg, Germany; 5 Division of Systems Biology of Signal Transduction, German Cancer Research Center, 69120 Heidelberg, Germany

**Keywords:** Biomarkers, Outcomes research, Translational research

## Abstract

Breakdown of synthesis, excretion and detoxification defines liver failure. Post-hepatectomy liver failure (PHLF) is specific for liver resection and a rightfully feared complication due to high lethality and limited therapeutic success. Individual cytokine and growth factor profiles may represent potent predictive markers for recovery of liver function. We aimed to investigate these profiles in post-hepatectomy regeneration. This study combined a time-dependent cytokine and growth factor profiling dataset of a training (30 patients) and a validation (14 patients) cohorts undergoing major liver resection with statistical and predictive models identifying individual pathway signatures. 2319 associations were tested. Primary hepatocytes isolated from patient tissue samples were stimulated and their proliferation was analysed through DNA content assay. Common expression trajectories of cytokines and growth factors with strong correlation to PHLF, morbidity and mortality were identified despite highly individual perioperative dynamics. Especially, dynamics of EGF, HGF, and PLGF were associated with mortality. PLGF was additionally associated with PHLF and complications. A global association-network was calculated and validated to investigate interdependence of cytokines and growth factors with clinical attributes. Preoperative cytokine and growth factor signatures were identified allowing prediction of mortality following major liver resection by regression modelling. Proliferation analysis of corresponding primary human hepatocytes showed associations of individual regenerative potential with clinical outcome. Prediction of PHLF was possible on as early as first postoperative day (POD1) with AUC above 0.75. Prediction of PHLF and mortality is possible on POD1 with liquid-biopsy based risk profiling. Further utilization of these models would allow tailoring of interventional strategies according to individual profiles.

## Introduction

Worldwide, about 1.4 million patients undergo a liver resection every year^1,2^. A growing subgroup of patients requires extended surgical procedures to achieve complete tumor clearance^3^. Morbidity and mortality rates for major hepatectomies remain high^4–6^. Post-hepatectomy liver failure (PHLF) is the most life-threatening complication with incidence varying from 1.2 to 32%^7–13^. Currently, treatment of PHLF is limited to supportive interventions against progressive dysregulation in the hepato-organic axis and no comparative trials are available^14^.

Patients would benefit from a prediction of their individual liver regenerative capacity. Data-driven mathematical models proved effects of cytokine and growth factor regulated regeneration networks of the liver remnant^15–20^. Emerging evidence suggests that signalling pathway markers can be used as individual signatures^21^. The association of cytokines and growth factors with liver regeneration potency is widely studied in animal models but clinical data are scarce^22,23^.

Therefore, we use a pragmatic approach by generating a time-dependent dataset of regeneration regulating cytokines and growth factor profiles based on liquid-biopsy samples from patients undergoing major liver resection. By consideration of this data along with pre- and perioperative clinical information and combination with three different mathematical modelling approaches, we generated an easily assessable, non-invasive, and inexpensive test option reliably predicting individual liver function recovery potency.

## Results

**Figure 1:**
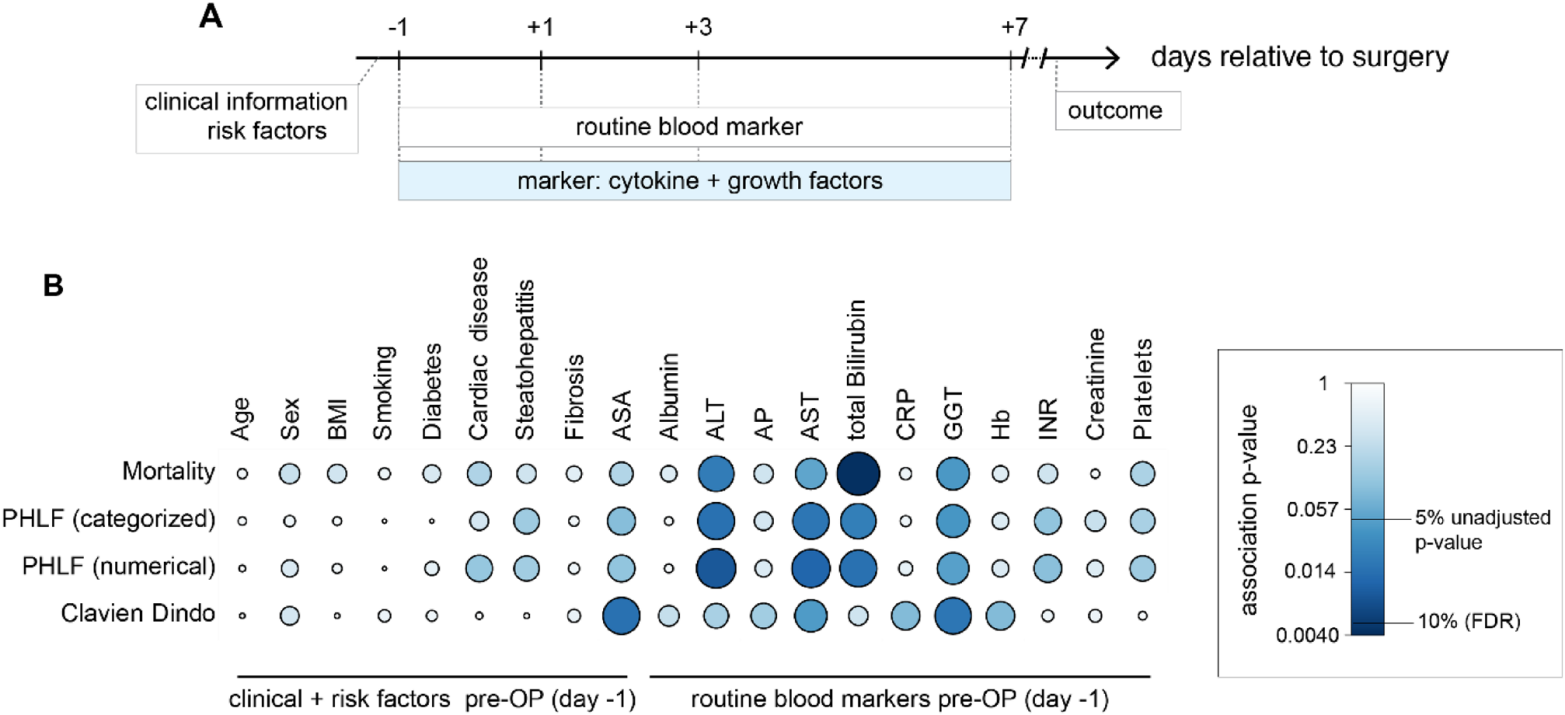
Study cohort overview of routine data and additional plasma marker. (**A**) Schematic overview of available data. (**B**) Pairwise-associations tests of routinely available preoperative data with four outcome parameters. Colors and size of the circle indicate the strength of association. The first nine columns correspond to general clinical information and known risk factors. The last 11 columns represent routine blood markers measured one day before surgery. The risk of mortality was significantly associated with the level of preoperative total bilirubin after adjusting for multiple testing (Kruskal–Wallis test unadjusted *p* value of 0.0033, FDR adj. *p* = 0.084).

The study course is summarized in Fig. 1A. Patient characteristics are provided in Table 1. Overall morbidity (Clavien-Dindo ≥ 3) was 31.8% and PHLF of any grade occurred in 34.1% of patients (n = 15). The median future liver remnant (FLR) to body weight ratio was 0.79% in patients with PHLF and 1.15% in patients without PHLF (unadjusted *p* = 0.053). The differences in PHLF, overall morbidity ≥ 3 or mortality rates for various indications for hepatectomy were not significant (*p* = 0.99; *p* = 0.39; *p* = 0.26). The median survival of the training cohort was 36 days (range 8–138).

**Table 1:** Patient characteristics.

Parameter	Entire cohort (n = 44)	Training cohort (n = 30)	Validation cohort (n = 14)	*p* values
	Median (range) N %	Median (range) N %	Median (range) N %	
**Gender**				0.738
Male	30 (68.2%)	21 (70%)	9 (64.3%)	
Female	14 (31.8%)	9 (30%)	5 (35.7%)	
Age (years)	64 (18–82)	61 (18–82)	69.5 (28–78)	0.246
**Hepatic resection**				
Right hepatectomy	17 (38.7%)	10 (33.3%)	7 (50%)	0.334
Left hepatectomy	14 (31.8%)	10 (33.3%)	4 (28.6%)	1.000
Right trisegmentectomy	10 (22.7%)	8 (26.7%)	2 (14.3%)	0.462
Left trisegmentectomy	3 (6.8%)	2 (6.7%)	1 (7.1%)	1.000
**Tumor entity**				0.001
Intrahepatic CC	13 (29.5%)	10 (33.3%)	3 (21.4%)	
Perihilar CC	7 (15.9%)	5 (16.7%)	2 (14.3%)	
HCC	9 (20.5%)	7 (23.3%)	2 (14.3%)	
CRCLM	8 (18.2%)	3 (10%)	5 (35.7%)	
Other malignant	5 (11.4%)	3 (10%)	2 (14.3%)	
Benign	2 (4.5%)	2 (6.7%)	0	
**Cofactors**				
Neoadjuvant Chemotherapy	13 (29.5%)	6 (20%)	7 (50%)	
BMI (kg/m^2^)	25.9 (15.9–37.2)	24.8 (15.9–36.3)	27 (17.8–37.2)	0.199
ASA (≧ 3)	20 (45.5%)	13 (43.3%)	7 (50%)	0.690
Fibrosis	36 (81.8%)	26 (86.7%)	10 (71.4%)	0.743
Steatohepatitis	22 (50%)	15 (34.1%)	7 (50%)	0.666
Cirrhosis	3 (6.8%)	3 (10%)	0	0.540
Diabetes	8 (18.2%)	5 (16.7%)	3 (21.4%)	0.695
Cardiac disease	20 (45.5%)	12 (40%)	8 (57.1%)	0.327
Renal disease	3 (6.8%)	1 (3.3%)	2 (14.3%)	0.234
**Volumetry**				
Body weight ratio FLR	0.908%	0.935%	0.834%	0.053
**Preoperative parameters**				
Serum bilirubin (mg/dl)	0.6 (0.2–11.2)	0.6 (0.2–11.2)	0.5 (0.3–1.4)	
AP (U/l)	147 (42–551)	149.5 (42–551)	144.5 (51–380)	
GGT (U/l)	153.5 (22–1915)	159.5 (22–1702)	134 (22–1915)	
AST (U/l)	32.5 (21–135)	31 (21–135)	39 (22–65)	
ALT (U/l)	37 (12–122)	33 (12–108)	41 (19–122)	
Platelets (1/nl)	232.5 (73–655)	260 (73–655)	227.5 (104–455)	
Albumin (g/l)	43.25 (31.6–50.2)	44.1 (31.6–50.2)	42.6 (37.2–49.6)	
Creatinine (mg/dl)	0.78 (0.49–1.43)	0.81 (0.52–1.4)	0.74 (0.49–1.43)	
**Complications Clavien Dindo**				0.105
No complications	9 (20.5%)	3 (10%)	6 (42.9%)	
Grade 1	6 (13.6%)	4 (13.3%)	2 (14.3%)	
Grade 2	8 (18.2%)	8 (26.7%)	0	
Grade 3	7 (15.9%)	4 (13.3%)	3 (21.4%)	
Grade 4	7 (15.9%)	6 (20%)	1 (7.1%)	
**PHLF ISGLS**				0.881
No PHLF	29 (65.9%)	20 (66.7%)	9 (64.3%)	
Grade A	4 (9.1%)	2 (6.7%)	2 (14.3%)	
Grade B	4 (9.1%)	2 (6.7%)	2 (14.3%)	
Grade C	7 (15.9%)	6 (20%)	1 (7.1%)	
**Mortality**	7 (15.9%)	5 (16.7%)	2 (14.3%)	1.000
Median Heidelberg Risk Score^25^ (Points)	3	3.5	3	
**Postoperative stay**				
ICU (days)	0 (0–36)	0 (0–36)	0 (0–13)	0.521
Intermediate care (days)	1 (0–99)	1 (0–99)	0.5 (0–58)	0.360
Total hospitalisation (days)	14 (5–138)	18.5 (5–138)	9.5 (7–97)	0.121

ICC, Intrahepatic colangiocarcinoma; ECC, extrahepatic colangiocarcinoma; HCC, Hepatocellular carcinoma.

Preoperative albumin was missing in 10 patients.

### Routine data and risk factors

Pre-operative routine blood parameters and clinical parameters were associated with the outcomes of interest in the training cohort. Excerpt from all 2319 associations are shown in Fig. 1B and Appendix—Table A1. Fourteen associations with unadjusted *p* < 5% were identified. After adjusting for multiple testing, only pre-operative total bilirubin above normal range was significantly associated with mortality (false discovery rate—FDR—adj. *p* = 0.084).

### Cytokines and growth factor levels vary distinctly before and after surgery

**Figure 2:**
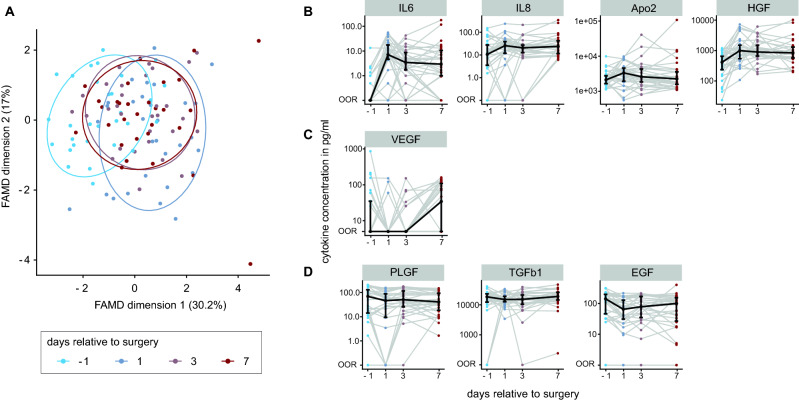
Cytokine and growth factor levels vary before and after surgery. (**A**) Factor analysis for mixed data on cytokine and growth factor data. The first dimension explains 30.2% of the variance and separates preoperative time point (day -1) from postoperative time points. 80% confidence intervals are drawn per time point. Every point represents one patient for one time point. (**B**) Time course of cytokines and growth factors relative to surgery showing increasing concentrations. (**C**) Time course of VEGF showing concentrations below the detection limit until POD7. (**D**) Time course of cytokines and growth factors with a slight decrease. Median and IQR (25% and 75% quantiles) are indicated in black. Each patient is connected by one grey line over the time points. OOR depicts out of range values.

Perioperative individual cytokine and growth factor dynamics were captured by longitudinal measurements performed at four time points per patient. To understand the global relation among the cytokines and growth factors a dimension reduction technique called factor analysis for mixed data (FAMD) was applied. The low-dimensional representation obtained from the factor analysis showed a separation of preoperative measurements (day -1) from postoperative measurements (POD1, 3 and 7) along the first dimension, which explains, by design, most of the variance (30.2%). Samples of day-1 and POD1 showed the largest separation indicating the biggest difference in combined expression patterns of all cytokines and growth factors (Fig. 2A).

Despite variability in time-trajectories of the cytokines and growth factors between individual patients, common expression patterns are visible. For four markers (IL-6, IL-8, Apo2 and hepatocyte growth factor, HGF) we observe an increased median concentration following surgery (Fig. 2B). IL-6 levels increased by 64% between day-1 and POD1 to a median level of 7.1 pg/ml. Beyond POD1 IL-6 levels decreased. Conversely, the median concentration of epidermal growth factor (EGF), transforming growth factor (TGF)-β1 and placental growth factor (PLGF) decreased slightly (Fig. 2D). Median concentrations of vascular endothelial growth factor (VEGF) remained below the detection limit until POD7 (Fig. 2C). This led us to investigate whether patients with similar trajectories are sharing outcome parameters.

### Clusters of cytokine and growth factor trajectories associate with outcome

Cluster analysis of cytokine and growth factor trajectories grouped those patients into patient clusters who share similar cytokine and/or growth factor time profiles. Next, we investigated whether patient clusters were associated with outcomes of interest. There were no differences regarding patients BMI, remnant liver volume, liver body weight ratio, blood loss, transfusion rate or histological features among the clustered patients. Trajectories of HGF or EGF differed among individuals who died after liver resection compared to those who survived. Furthermore, specific PLGF dynamics could be linked to mortality, morbidity, and PHLF (Appendix—Table A2).

**Figure 3:**
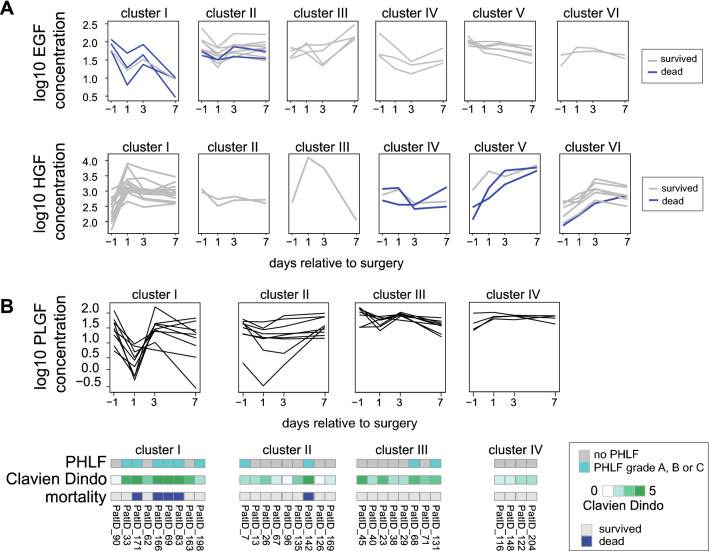
Clustering of growth factor trajectories related to clinical outcome parameter. Every cluster presents groups of patients with similar perioperative dynamics over time. (**A**) Concentrations over time for every patient shown in grey lines. Incidence of mortality indicated as a blue line. EGF and HGF were each split into six clusters I–VI, respectively. (**B**) Perioperative dynamics of PLGF split into four clusters (I–IV). Patient outcome parameters are depicted below according to their cluster assignment. Cluster I encompasses patients’ strong fluctuations and severe outcome.

Characteristic trajectories of EGF concentrations showed association with mortality (unadjusted *p* = 0.025). In Cluster I exhibiting EGF concentration drop on POD1 from similar preoperative levels three out of four of patients died. HGF trajectory clustering yielded the strongest association with 6 clusters linked to mortality (unadjusted *p* = 0.014). Cluster I represents HGF trajectory with favourable outcome for patients if HGF peaks on POD1 until it drops slightly by POD7 (Fig. 3A). PLGF trajectories associated with all investigated outcome parameters and fluctuations of PLGF concentration between day -1 to POD3 are higher in patients with PHLF, mortality, and complications than those with favourable outcomes. Cluster I of PLFG encompass patients with the strongest fluctuations that associate with fatal / severe outcomes. Conversely, cluster IV represents patients with stable PLGF levels and associates with favourable outcome (Fig. 3B). In summary, we could identify certain cytokine or growth factor behaviour over time which are associated with negative outcomes for the patients. Mostly, high fluctuations (cluster I for EGF and PLFG) are associated with negative outcomes.

### Global association network

**Figure 4:**
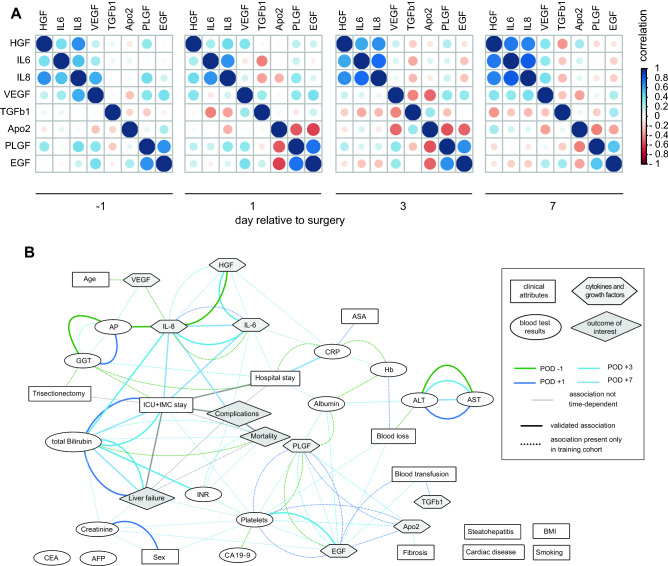
Timepoint dependent cytokine and growth factor concentrations and association among plasma proteins and clinical attributes. (**A**) Pairwise correlation of cytokine and growth factor concentrations separated by time points. (**B**) Global association network of known and novel study parameters. Oval nodes display the laboratory values, rectangular nodes the clinical information, hexagonal nodes cytokines or growth factors, and the diamond-shaped outcome parameter of interest. Every line indicates a significant association between two nodes. Dotted lines show association in training cohort data, solid lines associations validated in the validation data. PHLF numerical and categorized showed exact same interactions and were combined as PHLF for easier visualization.

Calculating time-point-specific association among the cytokines and growth factors, hepatocyte proliferation and angiogenesis promoting cytokines IL-6 and IL-8 revealed a high degree of association. HGF showed association with IL-6 and IL-8 at several time points indicating strong connection with the pro-regenerative response on POD3 + POD7. PLGF and EGF correlated over all time points linking stimulation of regeneration and hepatocyte proliferation (Fig. 4A, Appendix—Table A3).

To show the interdependence of biomarkers with clinical parameters time-point-specific associations were computed and visualized in a network (Fig. 4B). The procedure identified a total of 109 significant associations in the training cohort (dotted lines in Fig. 4B). Of these 34 could be replicated in the validation data (solid lines in Fig. 4B). Here, we describe the validated associations between cytokines or growth factors and clinical parameters. Measurements of IL-6 (POD7) were associated with length of ICU and IMC stay. IL-8 (POD7) correlated with occurrence of complications, prolonged ICU and IMC stay and total bilirubin. EGF (POD3) correlated with platelets. Platelets were identified as a hub in the network in the training cohort connecting biomarkers and clinical outcomes but this could not be replicated in the validation cohort. The risk of mortality correlated with complications. Liver failure was associated with prolonged ICU and IMC stay and with increased total bilirubin levels on POD1-POD7. Complications were associated with prolonged ICU and IMC stay. Patients with high bilirubin stayed longer in ICU and IMC and had impaired renal function (INR, POD3). CRP (POD7) correlated negatively with hospital stay. Among the validated associations total Bilirubin and length of ICU and IMC stay can be identified as hubs in the network with several validated associations to cytokines (IL-6, IL-8), lab values (INR) and critical patient-centered outcomes (complications, liver failure) (Fig. 4B, Appendix—Table A3).

### Predicting mortality and PHLF

Finally, we systematically probed the data set for predictive power and applied three statistical methods to predict the occurrence of complications, PHLF or mortality based on all available variables as potential predictors. All available variables consist of cytokine and growth factor measurements on 4 different time points, lab measurements on the same days and clinical parameters. All variables were regarded as individual, independent predictors in the respective models. For each measurement day, a separate model was calculated. Overall, the data has predictive power for mortality and occurrence of PHLF (See Appendix).

**Figure 5:**
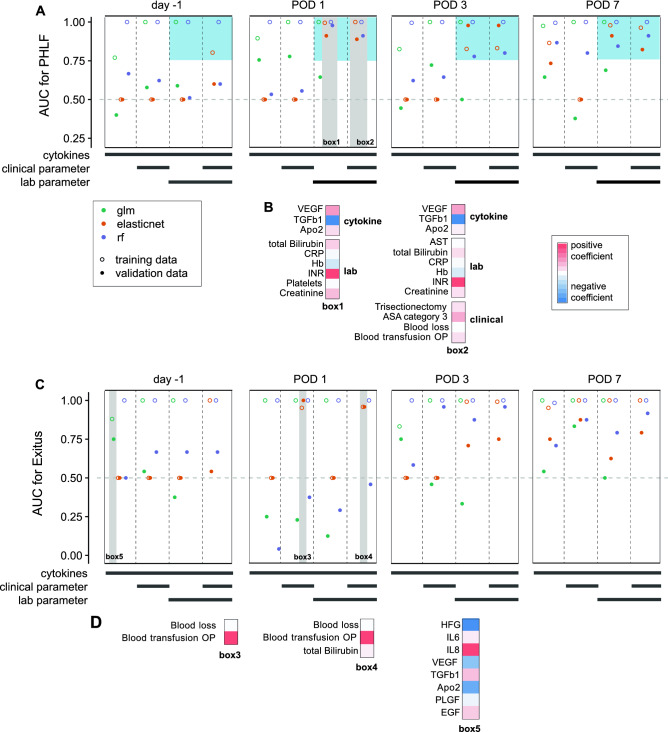
Predicting mortality and PHLF (**A**) AUC for predicting PHLF from different data sets. Data indicated on the x-axis consisted either of only cytokines, cytokine and clinical data, cytokine and routine lab data, or all three data. For each model, only data from one time point was used. Grey bars highlight AUC from training data (hollow dots) and validation data (solid dots blue box) from elastic net and random forest (orange and purple) close to 1 on POD 1. (**B**) Coefficients of the non-zero coefficients of the elastic net models in grey boxes 1 and 2 are displayed, color-coded by the respective positive or negative estimate. (**C**) AUC for predicting mortality from different data sets. Data indicated on the x-axis consisted either of only cytokines, cytokine and clinical data, cytokine and routine lab data, or all three data. For each model, only data from one time point was used. The validation AUC (solid shape) for some applied methods (highlighted with grey boxed) and some data sets (x-axis) is relatively close to 1 even for data from day -1 and POD 1. (**D**) Same as B for boxes 3, 4 and 5.

PHLF: whenever laboratory parameters were included into the set of predictors, elastic net, a regularized logistic regression model, and random forest, a machine learning algorithm, were able to predict PHLF with AUC values above 0.75 for POD1, 3 and 7 in the validation cohort (Fig. 5A). Already POD1, so only one day after surgery, yielded the best predictions of PHLF, thus we next investigated the selected parameter from the respective elastic net logistic regression model (Fig. 5A, box1, Fig. 5B). The regularized model selected variables based on their optimal predictive performance. The selected variables were VEGF, Apo2, total bilirubin, INR, and creatinine and they were assigned positive model coefficients. On the other hand, TGF-β1, CRP, Hb, and platelets were assigned with negative coefficients. To give an intuition of the meaning of the model coefficients, patients with low POD1 plasma levels of these factors have higher odds of developing PHLF while patients with low levels of VEGF, Apo2, total bilirubin, and creatinine have lower odds of developing PHLF. Comparing finally to the other good performing model, when all data was available for selection on POD1 (Fig. 5A, box2, Fig. 5B), the elastic net model selected trisectionectomy, ASA grade 3, blood loss, intraoperative blood transfusion among others with a positive coefficient. In summary, already on POD1, two of the chosen modelling approaches were able to determine a mathematical, multivariable model on the training data with good performance on the independent, previously unseen validation cohort. This was only possible if besides cytokines and growth factors also laboratory measurements, like INR, creatinine, total bilirubin, etc. were included as predictors in the multivariable models.

Mortality: two models reliably predicted mortality at POD1 (Fig. 5C,D), of which selected variables were blood loss during operation and intraoperative blood transfusion (box 3). The elastic net model using all three data sets predicts that patients with high intraoperative blood loss, the need of blood transfusion and high bilirubin on POD1 have higher odds of mortality (box 4, Fig. 5C,D). To investigate the contribution of all cytokines and growth factors non-regularized logistic regression was performed which does not select certain predictors but assigns weights to all variables in the model (box 5, Fig. 5C,D). Low values of HGF, VEGF and Apo2 as well as high levels of IL-8 prior to surgery were associated with higher odds of mortality (AUC = 0.75). In summary, the clinical variables concerning blood loss or transfusion are predictive for mortality in this data set and the validation data set. Nevertheless, already on the day before the surgery a standard logistic regression model could predict mortality on the unseen validation data reasonably well.

### Individual regenerative capacity and outcome

**Figure 6:**
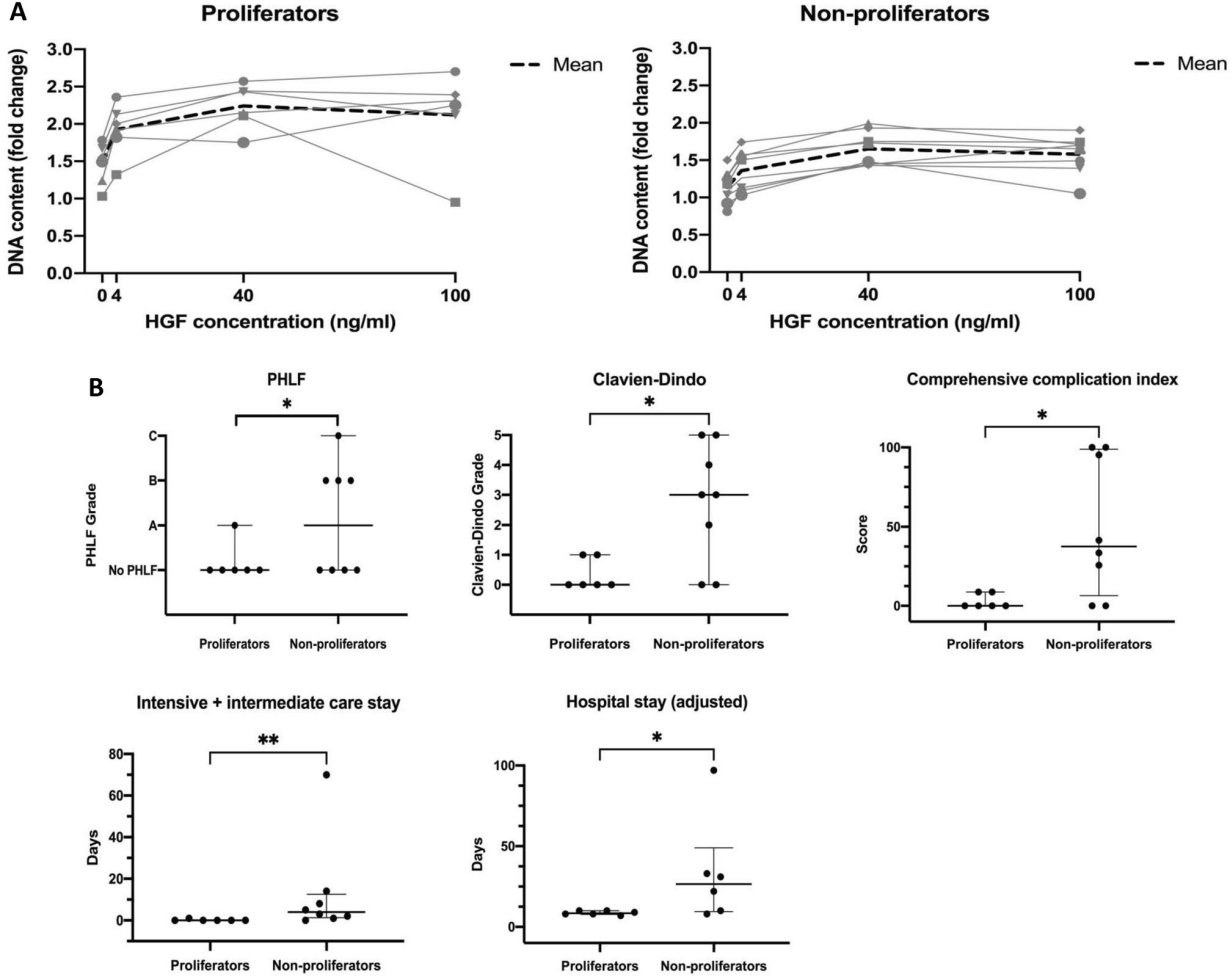
Individual regeneration capacity and clinical outcome. PHHs were isolated from the resected specimen of 14 patients from the validation cohort. (**A**) Proliferation response of PHHs. The maximal fold change of proliferation induced by HGF stimulation was chosen as grouping criteria. The cut-off was set at the fold change of two. Patients were grouped in proliferators and non-proliferators. (**B**) Association between proliferation response of PHHs and clinical outcome. Hospitals stay adjusted includes only patients without in-hospital mortality. Differences between groups were analysed by Mann–Whitney-U, Chi square and Fishers exact test. *p ≤ 0.05; **p ≤ 0.001.

Based on the maximal fold change of PHH proliferation under HGF stimulation patients were divided in proliferators and non-proliferators (Fig. 6A). Patients with good individual regenerative capacity of PHHs developed less complications (unadjusted *p* = 0.028), had a lower CCI (unadjusted *p* = 0.032), and showed shorter ICU (unadjusted *p* = 0.007) and hospital stays (unadjusted *p* = 0.046). Most importantly, proliferators developed less PHLF (unadjusted *p* = 0.040) (Fig. 6B).

## Discussion

Currently established protocols for diagnosis of PHLF have limitations identifying patients who will suffer from lethal complications. The combination of blood-based protein panel analysis and computational modelling provides the potential for generating objective quantitative information on functional liver recovery capacity and discovering high-risk patients with a non-invasive, fast and easily assessable test. This will allow an adaption of the surgical and interventional strategy and decision-making processes. The predictive power of the models is confirmed by analysis results of the individual proliferative capacity of primary hepatocytes.

Deficient inflammatory signalling and disturbed growth factor bioavailability lead to impaired liver regeneration^19^. We were able to detect and quantify various cytokine and growth factor signatures in longitudinal plasma samples. Several trajectories showed variable inter-individual changes, which emphasizes that regeneration is highly individual and dynamically controlled and not all factors respond alike across all times^19^. Unsupervised clustering of the trajectories per cytokine and growth factor, into optimal number of clusters displayed the inter-individual differences. By associating clusters with outcome parameters, we were able to document that specific dysregulations of hepato-regenerative and inflammatory mediators are associated with morbidity and mortality in the individual perioperative course. Notably, reproducible patterns over time for several growth factors associate with postoperative death, PHLF, and other complications. We do not propose to use the time series clustering as clinical predictors but merely present them to show the associations observed within out data set.

Correlation of all protein panel profiles, as well as clinical variables with each other and known markers for PHLF and postoperative mortality identified 109 significant associations. Of those, 34 were further validated in an independent prospective validation cohort. In this global association analysis involving 2319 individual comparisons, key factors that were tightly co-regulated belong to the innate and early pro-regenerative response pathways following liver damage. While interactions of IL-6, IL-8, and HGF are in line with the well-known evidence on liver regeneration, correlations of PLGF and EGF presumably reflect a new link to angiogenic stimulation of hepatic regenerative capacity^24,25^.

We intend to highlight the PLGF concentration. A pronounced variation over time was associated with severe complications and mortality. PLGF, a hypoxia-induced angiogenic factor plays a negligible role in physiological angiogenesis and is not required as a survival signal for the maintenance of quiescent vessels in healthy tissues^26^. The biological function of PLGF in neovascularization is controversial^27^. However, PLGF is expressed by human hepatocytes. In rodents, it is involved in liver regeneration and a time-dependent up-regulation of PLGF-mRNA has been shown after partial hepatectomy^28^. Via selective activation of Flt-1 and stimulation of sinusoidal endothelial cells, PLGF can influence liver regeneration^29^. In vitro, PLGF induces ERK1/2-signaling and promotes chemotaxis and proliferation of hepatic stellate cells^26^. PLGF prolongs the activation of PDGF-Ra and EGFR, possibly because of a transactivation of these receptors by FLT-1^30^. Our data indicates that a massive drop of PLGF concentration on POD1 associates with the severity of postoperative complications, as well as with mortality. We also detected a stable correlation of circulating PLGF with EGF levels over all time points and describe this for the first time in a human hepatectomy setting.

Additionally, clinical markers used for the definition of PHLF and impaired liver function—high bilirubin, low CRP, low albumin, and high creatinine—associated with a panel of four regeneration promoting mediators—IL-6, IL-8, HGF, and EGF—in the global association network. Among those, postoperative IL-6 and IL-8 levels correlated with an impaired outcome. To systematically test this data for predictive power, we used and compared various statistical models. Our modelling approach suggested that individual cytokine and growth factor profiles can be used to identify patients who will suffer from lethal complications following surgery. Low levels of HGF, VEGF, and Apo2 predict higher odds of mortality after liver resection while high levels of IL-8 seem to have protective effect. Our data provide the first evidence that a prediction of the individual regenerative capacity is possible with cytokine and growth profiling through a preoperatively taken serum sample.

Since PHLF is a major cause for mortality following liver resection we further investigated model-based prediction of PHLF. A reliable prediction of the occurrence of PHLF was possible already on POD1 when routine laboratory data were combined with cytokine and growth factor data. Data on cytokines and growth factors in the setting of post-hepatectomy liver failure are scarce^31–34^. An early detection of PHLF already at POD1 may be the key for minimizing the concomitant complications by an aggressive approach to support liver function and prevent acute fulminant (multi-)organ failure during the initial few days or a long-term functional deterioration over weeks. The suggested model-based approach might hereby be superior to the established 50–50 criteria by Balzan et al. and the ISGLS definition which both use the data of POD5 to diagnose PHLF^35^. Recent evidence suggests that the earliest possible resuscitation and supportive intervention for the liver might to reduce clinical consequences of PHLF^14^. Furthermore, individual regenerative capacity of PHHs was associated with the clinical outcome and especially PHLF. Determination of the individual proliferative capacity of patient hepatocytes could be an important new tool to define further therapeutic approaches in case of PHLF. Our proliferation experiment seems to indicate that regenerative capacity is largely determined by present constitution of the hepatocytes themselves and their consequent response to stimulation rather than the stimuli upon them. These results need to be validated further and translated into clinic.

The study presents some limitations, inherit to pilot works. The relatively small sample size warrants further internal and external validation, in particular pertaining to protein panel dynamics and to increase the sample size within the clusters. From the methodological perspective, the combined analysis of clinical, lab and biochemical data pose a challenge to the methods used, as these parameters are of mixed data types. We have chosen appropriate statistical tests for the nature of data and for subjecting resulting *p* values to a combined multiple testing strategy. In addition, we applied methods with linear (regression) and non-linear (random-forest) characteristics to probe the predictive power of the mixed data. Of note, we deliberately show performance on training and validation data side-by-side. We do not interpret model performance on training data alone; yet, such publications are available with potentially inflated, unvalidated results. The model performance on the training data is shown solely for potential comparison with other studies and is not considered an indicator of a good model. With the results and tested methods of our explorative study at hand, we will now be able to validate the results in a larger study cohort, with more shallow phenotyping and biochemical profiling to establish models that may be translated to clinical use.

## Conclusions

In conclusion, we found changes in circulating cytokines and growth factors of patients undergoing major liver resection that are clearly linked to the postoperative morbidity and mortality. With the demonstrated liquid biopsy-based approach we are able to predict potentially fatal postoperative clinical outcomes and the individual regenerative potential. This approach may allow a risk-adapted selection of patients. To strengthen the data-base of the model before incorporation in the clinical routine we aspire to include larger and more refined multi-center cohorts.

## Methods

### Study population

Forty-four patients undergoing elective open major liver resection (≥ 3 Couinaud segments, left or right hepatectomy as well as left or right trisectionectomy only) were included^36^. Inclusion criteria were: age ≥ 18 years, no prior liver resection; exclusion criteria: ALPPS procedure, laparoscopic resection, liver transplantation, resection due to hepatobiliary trauma, or resections combined with any other abdominal procedures. 30 patients (collected 2016–2018) were included in the training cohort and 14 patients (collected 2018–2019) were defined as the validation cohort. All patients signed an informed consent. Recruitment, blood/tissue sampling as well as blood and clinical data analysis were performed according to the Declaration of Helsinki and was approved by the Ethics Committee of Medical Faculty of Heidelberg University (S-557/2017). The surgical procedure was described earlier^37,38^. Indication was confirmed by multidisciplinary tumor board (Table 1). For volumetry details see Appendix.

### Blood and tissue sampling

Blood samples were collected on the day before surgery (day -1) and on postoperative day (POD) 1, 3, and 7. Primary human hepatocytes (PHHs) were isolated from the resected specimen according to a previously published protocol and stimulated with different concentrations of HGF and IL-6^39^. PHH proliferation was assessed by SybrGreen assay. For details see Appendix.

### Outcome parameter

PHLF was defined according to ISGLS^9^. PHLF was differentiated for statistical analysis in:

- categorical PHLF (PHLF cat.), defined as the postoperative development of PHLF with binary outcomes: yes or no
- as well as numerical PHLF (PHLF num.), defined as existing PHLF with grade A, B, or C (represented by numbers zero to three).

Postoperative complications were categorized according to Clavien-Dindo with grades 1 to 4^40^. In statistical modelling, complications were treated as a continuous variable with values 0 (no complications) to 5 (death) to include all patients for modelling. Overall morbidity was defined as any postoperative complication during the hospital stay and mortality as in-hospital death / Clavien-Dindo grade 5. The comprehensive complication index (CCI) was calculated for each patient^41^. The CCI is an established index calculated from Clavien-Dindo classification of complications, that tallies all complications in one patient, weighted by their severity, into a number on a continuous scale between 0 and 100. Value 0 depicts no complications, while 100 is assigned if the patient has died.

### Biomarker measurements

Multiplex bead-based immunoassays on a Bio-Plex® 200 Array Reader (Bio-Rad Laboratories, Munich, Germany) were used to measure HGF, EGF, TGF-β1, IL-6, IL-8, Apo2, PLGF and VEGF. For details see Appendix.

### Data processing

All data sets were subject to manual inspection and processing before the start of the analysis to ensure high data quality. For details see Appendix.

### Pairwise comparisons

Pairwise analysis of all gathered variables (2319 combinations) was performed on unprocessed data using rank-based methods where plasma protein values below detection limit were set to an arbitrary low value. Variables assessed at particular time points relative to liver resection were only compared with variables from the identical time points. Numerical variables were compared with Spearman’s rank correlation, numerical and factor variables were compared with Kruskal Wallis test. Two different factor variables with varying number of levels for each variable were compared using χ2-test. All comparisons were adjusted for multiple comparisons using the Benjamini–Hochberg procedure controlling the false-discovery rate (FDR) defined as the expected proportion of falsely rejected null hypotheses^42^. All presented *p* values are FDR-adjusted *p* values unless indicated otherwise. We set the threshold for significance of FDR adjusted *p* values to 10%. An association network for all variables was assembled, where nodes represent variables, which are connected by an edge when pairwise association showed significance (FDR < 10%). All pairwise comparisons were computed at once, thus, reported FDR adjusted *p* values are the result of one analysis including all variables. Analysis was performed using R version 3.5.3.

### Time series-clustering

Dissimilarities between time-series of one cytokine or growth factor were calculated using the “TSclust” R-package with the “CORT” method^43,44^. The “CORT” method combines pairwise temporal correlation between two observations and raw value behaviours through overall proximity of observations. Complete hierarchical clustering was performed on these dissimilarities and grouping in two, three, four, five and six clusters was assessed for the association to clinical outcome of patients. Associations to clinical outcomes were tested with Kruskal Wallis test and χ2-test. Only clinically relevant associations are shown. Analysis was performed using R version 3.5.3.

### Modeling

Three approaches were used:

1. logistic (binary outcomes are PHLF cat. and mortality) and linear non-regularized multivariable regression models (numeric outcomes are Clavien-Dindo and PHLF num.)
2. generalized linear models via penalized maximum likelihood with the elastic net penalty (α = 0.5) and leave-one-out cross-validation for the hyperparameter λ^45^. The response family was set to binomial for binary outcomes (PHLF cat. and mortality) and to Gaussian for numerical outcomes (Clavien-Dindo and PHLF num.). Calculations were performed in R, version 3.5.3, with the “glmnet” package^46^.
3. ensemble learning method random forest using classification trees for binary outcomes or regression trees for numerical outcomes^47^.

Models were evaluated using area under the curve (AUC) for binary outcomes and mean squared prediction error for numerical outcomes on training data and the unseen validation data. Per measurement day (day -1 and POD 1 to 7) separate models were estimated using only the data measured or available on that particular day. Cytokines and growth factor measurements were always used as predictors in all models. In addition to them, first clinical parameters were included as additional predictors. Next, cytokines and growth factor data were combined with lab measurements as additional independent variables without the clinical data. Finally, all three data sets (cytokines and growth factors, clinical and laboratory measurements) were used as independent variables together in multivariable regressions and as predictors in the random forest, respectively.

Model approach 1 (logistic and linear non-regularized multivariable regression models) was only applied if no more than two data sets were included as independent predictors to avoid overfitting. Model approach 2 (elastic net regularized regression) can handle more predictors and performs internal variable selection through the regularization parameter to avoid overfitting. Model approach 3 (random forest) has no limitation on the number of predictors. Each model was trained, e.g. estimated, on the training data. The resulting model was then evaluated on the previously unseen validation data to obtain an unbiased accuracy measure for each model.

### Association between proliferation response and clinical outcomes

Primary human hepatocytes were isolated from tissue of 14 patients, with 1 stemming from the main and 13 from the validation cohort, using an established two-step collagenase perfusion protocol^48^. In short, non-tumor liver tissue from patients was flushed with perfusion solution, then digested with a collagenase-containing solution. After digestion, the released hepatocytes were collected, washed and plated for culture. PHH were seeded in 6-well plates (Corning® BioCoat™ Collagen I 6-well Clear Flat Bottom TC-treated Multiwell Plate by Corning Life Sciences, Tewksbury, MA, USA) at a density of 150 000 cells per well in 1 ml adhesion medium (phenol red-free Williams E medium (Biochrom) supplemented with 10% (v/v) FCS (Gibco), 0.1 μM dexamethasone, 0.1% (v/v) insulin, 2 mM L-glutamine, 1% (v/v) penicillin/streptomycin (Gibco)) and incubated at 37 °C, 5% CO2 for 4 h. After washing with DPBS, cells were covered with starvation medium (phenol red-free Williams E medium (Biochrom) supplemented with 0.1 μM dexamethasone, 2 mM L-glutamine, 1% (v/v) penicillin/streptomycin) and incubated again for 16 to 20 h before stimulation.

The PHHs were stimulated over 48 h under 7 different conditions, in technical triplicates. The following substances were used for stimulation: recombinant human interleukin-6 (R&D Systems, Inc., Minneapolis, MN, USA) and recombinant human hepatocyte growth factor (R&D Systems, Inc., Minneapolis, MN, USA). Following concentrations were used: for HGF 4 ng/ml, 40 ng/ml and 100 ng/ml; for IL-6 10 ng/ml. Concentrations were combined to test single stimulus and combined-stimulus effects on proliferation as assayed for DNA content using Sybr®Green as previously described by Huard et al.^49^. Based on in-vitro proliferation, the patients were divided into two groups by the maximal fold change of proliferation induced by HGF. The cut-off was set at the fold change of two. The group with fold change above two were the “proliferators” while those patients with the value below were non-proliferators.

Associations with clinical outcomes were tested using Mann–Whitney-U test, χ2-test and Fischer’s exact test.

### Ethics approval and consent to participate

Recruitment, blood/tissue sampling as well as blood and clinical data analysis were performed according to the Declaration of Helsinki and was approved by the Ethics Committee of Medical Faculty of Heidelberg University (S-557/2017). All patients signed an informed consent form to participate.

## Supplementary Information

Supplementary Information.

## Data Availability

The datasets generated and analyzed during the current study are not publicly available due to internal confidentiality agreements but are available from the corresponding author, Prof. Dr. K. Hoffmann, on reasonable request.

## Abbreviations

ALPPS: Associating liver partition and portal vein ligation
ALT: Alanine transaminase
AP: Alkaline phosphatase
Apo2: Angiopoietin-2
ASA: American Society of Anaestesiologists
AST: Aspartate transaminase
AUC: Area under the curve
BMI: Body mass index
CC: Cholangiocarcinoma
CD: Clavien Dindo
CRCLM: Colorectal cancer liver metastasis
CRP: C-reactive protein
EGF: Epidermal growth factor
FDR: False discovery rate
FLR: Future liver remnant
GF: Growth factor
GGT: Gamma-glutamyl transpeptidase
Hb: Hemoglobin
HCC: Hepatocellular carcinoma
HGF: Hepatocyte growth factor
ICU: Intensive care unit
IFN: Interferon
IL-6: Interleukin-6
IL-8: Interleukin-8
INR: International normalized ratio
ISGLS: International Study Group of Liver Surgery
PHLF: Post-hepatectomy liver failure
PLGF: Placental growth factor
POD: Postoperative day
TGF-β1: Transforming growth factor beta1
TNF-α: Tumor necrosis factor alpha
VEGF: Vascular endothelial growth factor
