# Supplementary material for: Predicting liver regeneration following major resection
Source: Sci Rep. 2022 Aug 4;12:13396. doi: 10.1038/s41598-022-16968-9 (PMC9352754; doi:10.1038/s41598-022-16968-9)
Supplement: Supplementary file 1 — Supplementary Information. [file 41598_2022_16968_MOESM1_ESM.docx]

**Appendix**

**Material and Methods**

*Blood sampling and clinical data acquisition*

Blood was taken into a standard EDTA-tube. After centrifugation, the cell-free plasma was frozen at -80°C until further analysis. The following standard blood parameters were used for analysis: alanine transaminase (ALT, U/l), aspartate transaminase (AST, U/l), gamma-glutamyl transpeptidase (GGT, U/l), alkaline phosphatase (AP, U/l), total serum bilirubin (mg/dl), albumin (g/l), C-reactive protein (CRP, mg/l), hemoglobin (Hb, g/dl), international normalized ratio (INR), platelet count (1/nl) and creatinine (mg/dl) from all time points. Clinical information obtained included preoperative information on: age (years), gender, BMI (kg/m2), tobacco smoking, diabetes, cardiac disease, renal disease, calculated size of the future liver remnant (FLR, ml), and body-weight ratio of the calculated remnant (%); intra-operative information on blood loss (ml) and blood transfusion (yes/no); postoperative information on steatohepatitis, fibrosis and cirrhosis of the liver tissue as stated in the histopathological report.

*Liver volumetry*

Manual volumetry was performed by a board-certified radiologist with seven years of experience in abdominal imaging using the AquariusNET Viewer V.4.4.11.265 (TeraRecon Inc., Germany), based on axial portal venous phase cross-sectional images. If available, CT images were preferred over MR images. Extrahepatic bile ducts, the gallbladder, and surrounding extrahepatic vessels (e.g. extrahepatic portal vein) were excluded. Based on a preoperative CT/MRI scan, the preoperative total liver volume, total tumor volume, and the size of the future liver remnant were determined.

*Cytokine and Growth Factor Measurements*

Measurements were performed between 2017 – 2018 in three separate batches for the training cohort and in 2019 in one batch for the validation cohort. 30μl plasma per patient per time point were analyzed with Bio-Plex Pro Human Cancer Biomarker Assay (Apo-2, PLGF, EGF), Bio-Plex Pro Human Cytokine, Chemokine and Growth Factor Assay (HGF, VEGF, IL-6, IL-8), Bio-Plex Pro TGF-beta Assay (TGF-b1) from Bio-Rad Laboratories, Munich, Germany, and Milliplex MAP Human Angiogenesis/Growth Factor Magnetic Bead Panel- Cancer Multiplex Assay (Apo-2, PLGF, EGF) from Merck Millipore, Darmstadt, Germany in accordance to the corresponding manual, respectively.

*Data processing*

For modeling, the data was further processed: All incomplete clinical attributes were excluded from the modeling approaches (the only clinical attribute with missing data was steatohepatitis). Whenever standard blood markers were included in modeling, one patient and one standard blood marker (albumin) were excluded because of too many missing measurements (8 for the patient and 30% for albumin). For the remaining patients and markers, missing standard blood measurements (only 1-2% missingness) were imputed with mean values.

Plasma protein data was processed as follows: We chose to binarize plasma proteins with more than 50% of values beyond detection limit (only true for VEGF) into detected yes/no. For the remaining seven proteins, we first imputed missing values with the Gibbs sampler based imputation method “GSimp” and then log10-transformed them [1]. Next, we corrected the effect of the three batches in plasma protein data of the training cohort using the method “ComBat” which uses an empirical Bayes framework [2] We inspected the batch, corrected and imputed data with factor analysis for mixed data.

**Results**

*Table A1.*

List of the associations of clinical factors with outcome-parameters with unadjusted p-value below 5%.

| **Outcome** | **Clinical variable** | **Statistical test** | **p-value** | **FDR adj. p-value** |
| --- | --- | --- | --- | --- |
| Mortality | total Bilirubin | kruskal.test | 0.0033 | 0.084 |
| Mortality | ALT | kruskal.test | 0.019 | 0.19 |
| Mortality | GGT | kruskal.test | 0.037 | 0.27 |
| PHLF_num | ALT | cor.test | 0.008 | 0.13 |
| PHLF_num | AST | cor.test | 0.011 | 0.15 |
| PHLF_num | total Bilirubin | cor.test | 0.014 | 0.18 |
| PHLF_num | GGT | cor.test | 0.014 | 0.29 |
| PHLF_cat | ALT | kruskal.test | 0.014 | 0.18 |
| PHLF_cat | AST | kruskal.test | 0.014 | 0.18 |
| PHLF_cat | total Bilirubin | kruskal.test | 0.014 | 0.19 |
| PHLF_cat | GGT | kruskal.test | 0.014 | 0.27 |
| Clavien Dindo | AST | cor.test | 0.014 | 0.28 |
| Clavien Dindo | GGT | cor.test | 0.014 | 0.18 |
| Clavien Dindo | ASA | kruskal.test | 0.014 | 0.18 |

Clustering

*Table A2.*

Hits in time series clustering associated to clinical outcome with unadjusted p-value below 5%.

| **cytokine/GF** | **# of clusters** | **clinical outcome** | **p-value** | **test** |
| --- | --- | --- | --- | --- |
| HGF | 3,4,5,6 | mortality | 0.044, 0.016, 0.034, 0.014 | 𝟀^2^-test |
| PLGF | 3 | PHLF (categorical) | 0.039 | 𝟀^2^-test |
|  | 3,4,5 | PHLF (numeric) | 0.015, 0.032, 0.031 | kruskal-wallis test |
|  | 3,4,5 | Clavien Dindo | 0.050, 0.030, 0.032 | kruskal-wallis test |
|  | 3,5,6 | mortality | 0.022, 0.0098, 0.016 | 𝟀^2^-test |
| EGF | 6 | mortality | 0.025 | 𝟀^2^-test |
| IL6 - no hits |  |  |  |  |
| IL8 - no hits |  |  |  |  |
| TGFB - no hits |  |  |  |  |
| Apo2 - no hits |  |  |  |  |
| VEGF - not calculated because 0/1 coded | | |  |  |

Association network

*Table A3.*

Complete list of significant associations presented in the association network (FDR < 10% in the training cohort). Only adjusted p-values below 10% in the validation cohort are presented.

| **variable 1** | **variable 2** | **timepoint** | **test** | **adjusted p-value training cohort** | **adjusted p-value validation cohort** |
| --- | --- | --- | --- | --- | --- |
| Age | VEGF | -1 | cor.test | 0.021 |  |
| **Hospital stay** | **ICU+IMC stay** | **none** | **cor.test** | **0.0071** | **0.074** |
| Hospital stay | Complications | none | cor.test | 0.0059 |  |
| Hospital stay | IL-6 | 7 | cor.test | 0.078 |  |
| Hospital stay | GGT | -1 | cor.test | 0.079 |  |
| Hospital stay | GGT | 3 | cor.test | 0.078 |  |
| Hospital stay | Albumin | 7 | cor.test | 0.053 |  |
| Hospital stay | CRP | -1 | cor.test | 0.078 |  |
| **Hospital stay** | **CRP** | **7** | **cor.test** | **0.084** | **0.074** |
| **ICU+IMC stay** | **Complications** | **none** | **cor.test** | **0.001** | **0.074** |
| **ICU+IMC stay** | **PHLF_num** | **none** | **cor.test** | **0.001** | **0.027** |
| **ICU+IMC stay** | **IL-6** | **7** | **cor.test** | **0.038** | **0.058** |
| **ICU+IMC stay** | **IL-8** | **7** | **cor.test** | **0.083** | **0.024** |
| ICU+IMC stay | GGT | -1 | cor.test | 0.084 |  |
| **ICU+IMC stay** | **Bilirubin** | **1** | **cor.test** | **0.012** | **0.073** |
| **ICU+IMC stay** | **Bilirubin** | **3** | **cor.test** | **0.078** | **0.037** |
| **ICU+IMC stay** | **Bilirubin** | **7** | **cor.test** | **0.037** | **0.022** |
| ICU+IMC stay | Platelets | 7 | cor.test | 0.078 |  |
| Complications | PHLF_num | none | cor.test | 0.0059 |  |
| Complications | IL-6 | 7 | cor.test | 0.037 |  |
| **Complications** | **IL-8** | **7** | **cor.test** | **0.078** | **0.029** |
| Complications | INR | 7 | cor.test | 0.082 |  |
| **PHLF_num** | **Bilirubin** | **1** | **cor.test** | **0.022** | **0.022** |
| **PHLF_num** | **Bilirubin** | **3** | **cor.test** | **0.082** | **0.022** |
| **PHLF_num** | **Bilirubin** | **7** | **cor.test** | **0.015** | **0.0052** |
| PHLF_num | Platelets | 7 | cor.test | 0.017 |  |
| PHLF_num | Kreatinin | 7 | cor.test | 0.078 |  |
| Blood loss | EGF | 7 | cor.test | 0.012 |  |
| Blood loss | ALT | -1 | cor.test | 0.02 |  |
| Blood loss | Hb | 1 | cor.test | 0.067 |  |
| Blood loss | Platelets | 7 | cor.test | 0.065 |  |
| CA 19-9 | Platelets | -1 | cor.test | 0.084 |  |
| **HGF** | **IL-6** | **3** | **cor.test** | **0.022** | **0.073** |
| HGF | IL-6 | 7 | cor.test | 0.0007 |  |
| **HGF** | **IL-8** | **-1** | **cor.test** | **0.0062** | **0.022** |
| HGF | IL-8 | 3 | cor.test | 0.0062 |  |
| HGF | IL-8 | 7 | cor.test | 0.0022 |  |
| HGF | CRP | 7 | cor.test | 0.082 |  |
| IL-6 | IL-8 | -1 | cor.test | 0.078 |  |
| IL-6 | IL-8 | 1 | cor.test | 0.0062 |  |
| **IL-6** | **IL-8** | **3** | **cor.test** | **0.0002** | **0.022** |
| **IL-6** | **IL-8** | **7** | **cor.test** | **0.0000** | **0.016** |
| IL-6 | CRP | 7 | cor.test | 0.031 |  |
| IL-8 | VEGF | -1 | cor.test | 0.031 |  |
| **IL-8** | **AP** | **-1** | **cor.test** | **0.082** | **0.013** |
| **IL-8** | **Bilirubin** | **3** | **cor.test** | **0.091** | **0.085** |
| VEGF | AP | 3 | cor.test | 0.078 |  |
| Apo-2 | PLGF | 1 | cor.test | 0.084 |  |
| Apo-2 | PLGF | 3 | cor.test | 0.078 |  |
| Apo-2 | EGF | 1 | cor.test | 0.033 |  |
| Apo-2 | Platelets | 1 | cor.test | 0.078 |  |
| Apo-2 | Platelets | 3 | cor.test | 0.084 |  |
| PLGF | EGF | -1 | cor.test | 0.0071 |  |
| PLGF | EGF | 1 | cor.test | 0.0003 |  |
| PLGF | EGF | 3 | cor.test | 0.0059 |  |
| PLGF | EGF | 7 | cor.test | 0.084 |  |
| PLGF | Albumin | -1 | cor.test | 0.038 |  |
| PLGF | Albumin | 3 | cor.test | 0.026 |  |
| PLGF | Platelets | -1 | cor.test | 0.082 |  |
| PLGF | Platelets | 1 | cor.test | 0.037 |  |
| PLGF | Platelets | 7 | cor.test | 0.099 |  |
| EGF | Albumin | 3 | cor.test | 0.091 |  |
| EGF | Platelets | 1 | cor.test | 0.078 |  |
| **EGF** | **Platelets** | **3** | **cor.test** | **0.084** | **0.063** |
| EGF | Platelets | 7 | cor.test | 0.0082 |  |
| EGF | Creatinine | 7 | cor.test | 0.078 |  |
| **AST** | **ALT** | **-1** | **cor.test** | **0.0033** | **0.016** |
| AST | ALT | 1 | cor.test | 0.0000 |  |
| AST | ALT | 3 | cor.test | 0.0006 |  |
| **AST** | **ALT** | **7** | **cor.test** | **0.021** | **0.039** |
| ALT | Albumin | 3 | cor.test | 0.099 |  |
| **AP** | **GGT** | **-1** | **cor.test** | **0.0000** | **0.0036** |
| **AP** | **GGT** | **1** | **cor.test** | **0.0005** | **0.067** |
| AP | GGT | 3 | cor.test | 0.039 |  |
| AP | GGT | 7 | cor.test | 0.022 |  |
| **Bilirubin** | **INR** | **3** | **cor.test** | **0.0071** | **0.067** |
| Bilirubin | Creatinine | 7 | cor.test | 0.078 |  |
| Albumin | Hb | -1 | cor.test | 0.073 |  |
| CRP | Hb | -1 | cor.test | 0.0062 |  |
| CRP | Hb | 7 | cor.test | 0.087 |  |
| INR | Platelets | 3 | cor.test | 0.028 |  |
| Platelets | Creatinine | 7 | cor.test | 0.022 |  |
| ICU+IMC stay | Trisectionectomy | none | kruskal.test | 0.026 |  |
| ICU+IMC stay | Mortality | none | kruskal.test | 0.091 |  |
| **ICU+IMC stay** | **PHLF_cat** | **none** | **kruskal.test** | **0.010** | **0.056** |
| **Complications** | **Mortality** | **none** | **kruskal.test** | **0.025** | **0.074** |
| Complications | PHLF_cat | none | kruskal.test | 0.038 |  |
| PHLF_num | Mortality | none | kruskal.test | 0.0069 |  |
| **PHLF_num** | **PHLF_cat** | **none** | **kruskal.test** | **0.0001** | **0.0099** |
| TGF-β1 | Blood transfusion | 1 | kruskal.test | 0.091 |  |
| Apo-2 | Fibrosis | 7 | kruskal.test | 0.084 |  |
| EGF | Blood transfusion | 1 | kruskal.test | 0.075 |  |
| EGF | Blood transfusion | 7 | kruskal.test | 0.078 |  |
| GGT | Trisectionectomy | -1 | kruskal.test | 0.078 |  |
| Bilirubin | Mortality | -1 | kruskal.test | 0.084 |  |
| Bilirubin | Mortality | 3 | kruskal.test | 0.078 |  |
| Bilirubin | Mortality | 7 | kruskal.test | 0.078 |  |
| **Bilirubin** | **PHLF_cat** | **1** | **kruskal.test** | **0.047** | **0.056** |
| **Bilirubin** | **PHLF_cat** | **3** | **kruskal.test** | **0.099** | **0.049** |
| **Bilirubin** | **PHLF_cat** | **7** | **kruskal.test** | **0.027** | **0.022** |
| CRP | ASA | 1 | kruskal.test | 0.053 |  |
| INR | Mortality | 3 | kruskal.test | 0.047 |  |
| INR | Mortality | 7 | kruskal.test | 0.037 |  |
| Platelets | Gender | 7 | kruskal.test | 0.078 |  |
| Platelets | PHLF_cat | 7 | kruskal.test | 0.031 |  |
| **Creatinine** | **Gender** | **1** | **kruskal.test** | **0.091** | **0.073** |
| Creatinine | Gender | 7 | kruskal.test | 0.079 |  |
| Creatinine | PHLF_cat | 7 | kruskal.test | 0.084 |  |
| Mortality | PHLF_cat | none | chisq.test | 0.083 |  |

Modeling

Figure A1.


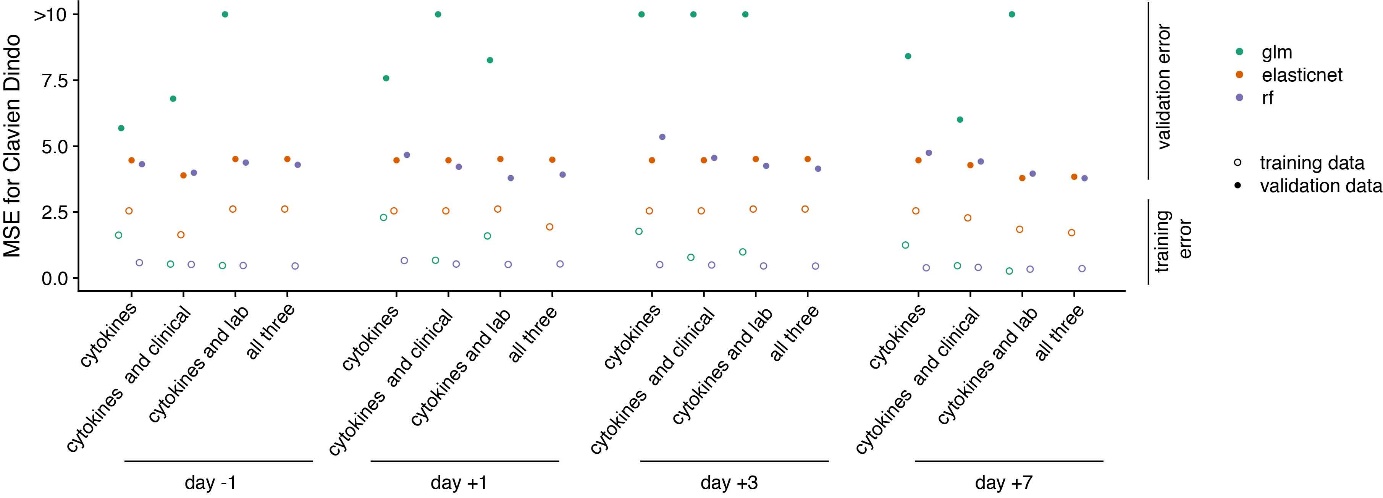


Prediction error (MSE = mean squared prediction error) for predicting Clavien-Dindo from different data sets. Data indicated on the x-axis consisted either of only cytokines, cytokine and clinical data, cytokine and routine lab data, or all three data. For each model, only data from one time point was used. The validation error (solid shape) is consistently and for all applied methods (indicated with various colors) and all data sets higher than the training error (hollow shape).

Reference for appendix:

[1] Wei, R., Wang, J., Jia, E., Chen, T., Ni, Y., & Jia, W. (2018). GSimp: A Gibbs sampler based left-censored missing value imputation approach for metabolomics studies. PLoS computational biology, 14(1), e1005973. <https://doi.org/10.1371/journal.pcbi.1005973>

[2] Zhang, Y., Jenkins, D.F., Manimaran, S. et al. Alternative empirical Bayes models for adjusting for batch effects in genomic studies. BMC Bioinformatics 19, 262 (2018). <https://doi.org/10.1186/s12859-018-2263-6>
